# Psychosocial Work Environment and Musculoskeletal Symptoms among 21-Year-Old Workers: A Population-Based Investigation (2011-2013)

**DOI:** 10.1371/journal.pone.0130010

**Published:** 2015-06-15

**Authors:** Sara Lourenço, Filomena Carnide, Fernando G. Benavides, Raquel Lucas

**Affiliations:** 1 EPIUnit—Institute of Public Health, University of Porto, Porto, Portugal; 2 Faculty of Human Kinetics, University of Lisbon, Cruz Quebrada—Dafundo, Portugal; 3 Research Centre in Occupational Health, Pompeu Fabra University, Barcelona, Spain; 4 Department of Clinical Epidemiology, Predictive Medicine and Public Health, University of Porto Medical School, Porto, Portugal; University of Pennsylvania, UNITED STATES

## Abstract

**Background:**

The current labour market is becoming more flexible and informal, with job insecurity selectively affecting young workers. However, the role of these increasing adverse psychosocial working conditions on health outcomes remains little known among newly employed workers.

**Objective:**

To estimate the associations between psychosocial work environment and musculoskeletal outcomes (widespread pain syndrome features and regional pain) in a population-based sample of young workers.

**Methods:**

Cross-sectional data from workers aged 21 years were collected during the third wave of the EPITeen cohort study (2011-2013; n=650). The Job Content Questionnaire was used to characterize the psychosocial work environment according to the demand-control-support model. Data on pain and non-pain dimensions of the widespread pain syndrome (Fibromyalgia Survey Questionnaire) as well as on regional musculoskeletal pain (Nordic Musculoskeletal Questionnaire) were also collected. Crude and adjusted odds ratios (OR) with 95% confidence intervals (95% CI) were computed using logistic regression and all estimates were adjusted for sex, education and occupational biomechanical demands.

**Results:**

Job insecurity was significantly associated to the non-pain dimension of the widespread pain syndrome (adjusted OR [95% CI]=1.51 [1.08, 2.12]). Young workers with strain jobs were significantly more likely to report high levels of non-pain symptoms when compared with those with no-strain jobs and this effect was even stronger when social support was added to the main exposure: workers with strain jobs and low social support had twice the odds of reporting high levels of non-pain features than those with high strain but high social support jobs (adjusted OR=1.86, 95% CI: 1.04, 3.31). These significant associations were not observed when widespread pain or multisite regional pain were the outcomes.

**Conclusion:**

In the beginning of professional life, high strain jobs were associated to non-pain complaints, especially when the work environment provided also low social support.

## Introduction

Occupation is an important determinant of health in general and its overall positive or negative effect on well-being results from the interaction between individual characteristics and work-related features, the latter comprising biomechanical, psychosocial and sociological axes [1, 2].

The impact of psychosocial work environment on health is particularly relevant under the current change of the labour market [3, 4], where jobs become more flexible [precarious, temporary, part-time], informal [unregulated, home based, non-standard work arrangements] and unstable/insecure [5]. Since young adults in the beginning of professional life have been remarkably affected by these new working conditions [6, 7], having up to date information on how the current psychosocial environment of work affects the health of newly employed workers is of major relevance [8, 9].

Classic biomedical approaches to the aetiology of musculoskeletal conditions have shown that mechanical exposures at work are among the main causes of muscle and joint complaints [10] and that such effect is observable since the early stages of professional life [11]. However, beyond the biomedical focus on work-related musculoskeletal disorders captured through the assessment of regional pain [12, 13], psychosocial dimensions have the potential to influence the occurrence of widespread pain syndrome features, including both its pain and non-pain components [14]. This hypothesis is supported by findings showing that workplaces characterized by high strain, low peer-support, conflicting tasks and/or stressful demands contribute importantly to a number of other physical and mental health conditions, namely cardiovascular diseases [15, 16] and clinical depression [17].

Most existing evidence of the impact of psychosocial work environment on musculoskeletal health has originated from long-term workers [18], where the potential for prevention is dramatically reduced [19] and where the typology of occupations does not reflect the emerging labour market. Research in the beginning of professional life is fairly sparse, mainly restricted to specific occupational groups [20–22] and commonly concerned with the impact of work-related psychosocial factors on already established health conditions [23].

Therefore, using data collected in a population-based sample of newly employed young workers with a wide spectrum of jobs (2011–2013), we aimed to estimate the associations between psychosocial work environment and musculoskeletal outcomes (widespread pain syndrome features and regional pain).

## Methods

### Ethics statement

The study protocol was approved by the Ethics Committee of University Hospital de São João (Porto) and by the Portuguese Authority of Data Protection and was carried out in accordance with the principles of the 1964 Declaration of Helsinki. Written informed consent was obtained from all participants.

### Participants

This investigation uses cross-sectional data collected in the period of 2011–2013 from young adults of the Epidemiological Health Investigation of Teenagers in Porto (EPITeen). This cohort was first assembled during the 2003/2004 school year, when all the public and private schools in Porto (Portugal) that provided teaching to adolescents born in 1990 were approached. We identified 2787 eligible adolescents, of whom 2159 (77.5%) agreed to participate. In 2007/2008, the initially recruited sample was re-evaluated and 783 students born in the same year but who moved to Porto after 2003/2004 were additionally recruited to the cohort. Sampling procedures and detailed methods have already been described elsewhere [24].

Between 2011 and 2013, 1761 (of the 2942 eligible) individuals attended the 21 years old follow-up. These were similar to participants lost to follow-up regarding a wide spectrum of characteristics such as sex, body mass index and smoking behaviour at 13 years of age. However, subjects attending follow-up had higher parental education (mean schooling years: 11.4 vs. 9.2, p<0.001).

**Fig 1:**
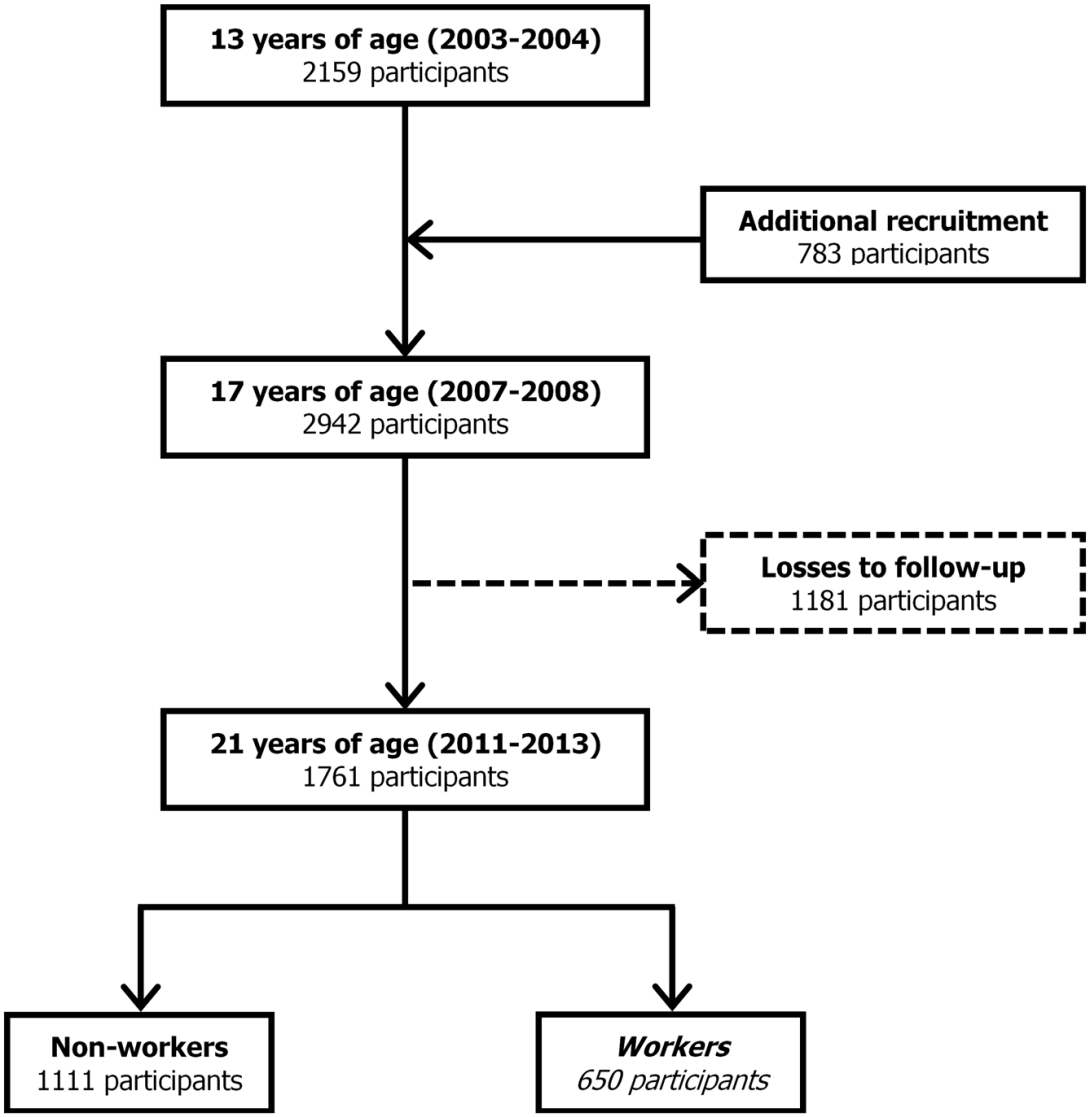
Flowchart illustrating the selection of workers from the EPITeen cohort (n = 650).

Among the 1761 young adults assessed at 21 years of age, 650 (36.9%) had worked during at least one month in the preceding year (13.3% were unemployed in the moment of evaluation) and constituted the final sample for this investigation (Fig 1). Neither psychosocial work environment perception (exposures) nor musculoskeletal complaints (outcomes) were significantly different between employed and unemployed workers. The remaining participants (n = 1111) had not worked in the preceding year (more than 80% full-time students).

Workers with incomplete responses in some of the exposure variables were considered in the analyses. Our methodological option of not excluding these subjects was supported by the absence of significant differences between workers with complete and incomplete responses in crucial characteristics such as sex distribution, education and occupational biomechanical demands (i.e., missing values were probably at random).

### Data collection

#### Socioeconomic and biomechanical factors

As educational level has been the most accurate indicator to assess the effect of socioeconomic position on health outcomes in the Portuguese population [25], formal education was recorded as completed years of schooling and used as proxy of socioeconomic position. Individual monthly income was gathered in euros and workers were classified using the 500 euros cut-off (the Portuguese minimum monthly wage was 485 euros between 2011–2013). Data on working regime (full- or part-time) as well as duration of the present job (in months) were also collected.

Jobs were classified according to the International Standard Classification of Occupations-88 [26] and grouped as follows: high-skilled clerical (legislators, senior officials, managers, professionals, technicians and associated professionals), intermediate-skilled clerical (clerks), low-skilled clerical (service workers and shop/market sales workers) and low and high-skilled manual (armed forces, skilled agricultural and fishery workers, craft and related trade workers, plant and machine operators and assemblers and elementary jobs).

Ten different occupational biomechanical factors (sitting posture, computer use, whole body vibrations, vibration tools handling, precision work, repetition, overhead and kneeling work, bending/rotation, manual materials handling) were also collected. Four patterns of exposure to occupational biomechanical factors were obtained in our sample of workers (n = 650) using the latent class analysis: low, sitting, repetitive and asymmetric and high and vibrational demands. A more detailed description on methods used to obtain the patterns of occupational biomechanical demands has been described elsewhere [27].

#### Psychosocial work environment

Psychosocial work environment of the job performed for the longest period of time in the past 12 months was assessed using the self-administered Job Content Questionnaire [28, 29], which allows for the computing of three main dimensions: decision latitude (sum of skill discretion and decision-making authority scales), psychological demands and social support (sum of supervisor and co-worker support scales). Perceived job insecurity was measured using three additional items of Job Content Questionnaire (“how steady is your work”, “during the past year, how often were you in a situation where you faced job loss or layoff” and “my job security is good”). Considering the whole sample of workers, the mean scores in each dimension were obtained. Participants were then grouped as above (higher) or below (lower) the sample mean score in each of the abovementioned dimensions.

Taking into account the demand-control-(support) model [30], two different strategies were used to assess the role of job strain on musculoskeletal complaints. In the first strategy (demand-control), data on decision latitude and psychological demands were used and workers were classified as having: a) no-strain jobs (low decision latitude & low psychological demands; high decision latitude & low psychological demands; high decision latitude & high psychological demands); b) strain jobs (low decision latitude & high psychological demands). In the second strategy (demand-control-support), social support was included and workers were grouped as having: a) no-strain & high social support jobs; b) no-strain & low social support jobs; c) strain & high social support jobs; and d) strain & low social support jobs.

#### Musculoskeletal outcomes

Widespread pain syndrome features were assessed using the Fibromyalgia Survey Questionnaire which allows for the quantification of the severity of a wide range of pain and non-pain symptoms [31]. Non-pain symptoms were measured using the symptom severity score (range: 0–12), composed by the following items: fatigue, trouble thinking or remembering, waking up tired/unrefreshed, pain or cramps in lower abdomen, depression and headache/migraine. Pain symptoms were evaluated using the widespread pain index (range: 0–19) that inquires about the presence of pain in the preceding seven days in nineteen anatomical sites (jaws, shoulders, upper arms, lower arms, hips, upper legs, lower legs, neck, chest, upper back, lower back and abdomen). Median scores in pain and non-pain dimensions were calculated in the whole sample (n = 1761). Workers were then grouped as above (higher) or below (lower) the sample median scores in both dimensions of the widespread pain syndrome. More detailed information on widespread pain syndrome features obtained in the EPITeen cohort has been described elsewhere [32].

Regional musculoskeletal pain was assessed using the Nordic Musculoskeletal Questionnaire [33] which evaluates the presence of acute pain in the preceding 12 months in nine different anatomical sites: neck, shoulders, elbows, wrists/hands, upper back, lower back, hips/thighs/buttocks, knees and ankles/feet. The sum of the number of painful regions was computed (range: 0–9) and workers were classified as having multisite pain if two or more anatomical regions were reported as painful.

### Statistical analysis

Sample characteristics are presented as counts and proportions for all the categorical variables.

In order to estimate the associations between psychosocial work environment (decision latitude, psychosocial demands, social support and job insecurity) and musculoskeletal outcomes (non-pain symptoms, widespread pain and multisite regional pain), logistic regression models were fitted using as reference the category with the expected lowest risk for the outcomes considered. Regarding the demand-control-(support) model analyses, logistic regression models were fitted using as reference workers with no-strain jobs (demand-control) or those with no-strain & high social support jobs (demand-control-support). Based on previous evidence, confounders of the associations between psychosocial work environment and musculoskeletal outcomes were defined *a priori*. Crude and adjusted odds ratios (OR) with 95% confidence intervals (95% CI) were computed and all estimates were adjusted for sex, education and occupational biomechanical demands.

Because manual workers are expected to be different from clerical workers in relation to psychosocial work environment exposure [34], a sensitivity analysis restricted only to clerical workers was additionally conducted.

Statistical analyses were performed using Stata version 11.2 for Windows (Stata Corp. LP, College Station, Texas, USA).

## Results

Table 1 summarises the characteristics of the 650 young workers from the EPITeen cohort. More than half of participants were women (51.2%), 55.5% had 12 or less completed schooling years and 64.4% had an individual income equal or below 500 euros. In relation to the occupational characteristics, 51.1% of workers reported to have part-time jobs, 69.1% had been working for 12 or less months and 56.2% had jobs mainly characterized by low biomechanical demands (low overall demands and sitting demands). The vast majority of participants (86.8%) had clerical jobs.

**Table 1:** Socioeconomic and occupational characteristics of young workers (n = 650; EPITeen cohort, 2011–2013).

Workers^a^ (n = 650)	Total
	n (%)
**Sex**	
Female	333 (51.2)
Male	317 (48.8)
**Education (formal schooling)**	
≤9 years	77 (11.8)
10–12 years	284 (43.7)
>12 years	289 (44.5)
**Monthly individual income**	
≤500 euros	417 (64.4)
>500 euros	231 (35.6)
**Working regime**	
Full-time	298 (48.9)
Part-time	311 (51.1)
**Work duration (lifetime)**	
<6 months	169 (28.9)
6–12 months	235 (40.2)
>12 months	181 (30.9)
**Work type**	
High skilled-clerical	146 (25.1)
Intermediate skilled-clerical	60 (10.3)
Low skilled-clerical	299 (51.4)
High and low skilled-manual	77 (13.2)
**Occupational biomechanical demands**	
Low	182 (28.0)
Sitting	183 (28.2)
Repetitive and asymmetric	141 (21.7)
High and vibrational	144 (22.2)
**Psychosocial work environment**	
Low strain	153 (26.3)
Active	144 (24.7)
Passive	129 (22.1)
High strain	157 (26.9)

^a^ Sample size is not constant due to missing data in monthly individual income (n = 2), working time (n = 41), work duration (n = 65), work type (n = 68) and psychosocial work environment (n = 67).

Table 2 presents the crude and adjusted associations between psychosocial work environment characteristics (decision latitude, psychological demands, social support and job insecurity) and high levels of non-pain symptoms and widespread pain as well as multisite regional pain. Results are presented for all workers and restricted to those with clerical jobs.

**Table 2:** Psychosocial work environment: associations with musculoskeletal outcomes (non-pain symptoms, widespread pain and multisite regional pain) considering all workers and restricting to clerical workers (EPITeen cohort, 2011–2013).

			Non-pain symptoms			Widespread pain			Regional pain (2 or more sites)		
		Total^a^	High^b^	Crude	Adjusted^d^	High^c^	Crude	Adjusted^d^	Multisite	Crude	Adjusted^d^
		n (%)	n (%)	OR (95% CI)	OR (95% CI)	n (%)	OR (95% CI)	OR (95% CI)	n (%)	OR (95% CI)	OR (95% CI)
**ALL WORKERS**	**Decision latitude**										
	High	302 (50.0)	110 (36.4)	1	1	122 (40.4)	1	1	196 (64.9)	1	1
	Low	302 (50.0)	143 (47.4)	1.57 (1.13, 2.17)	1.50 (1.06, 2.11)	125 (41.4)	1.04 (0.75, 1.44)	1.00 (0.72, 1.39)	200 (66.2)	1.06 (0.76, 1.48)	1.01 (0.72, 1.43)
	**Psychological demands**										
	Low	305 (48.8)	129 (42.3)	1	1	127 (41.6)	1	1	213 (69.8)	1	1
	High	320 (51.2)	140 (43.8)	1.06 (0.77, 1.46)	1.08 (0.77, 1.51)	133 (41.6)	1.00 (0.73, 1.37)	0.96 (0.69, 1.34)	201 (62.8)	0.73 (0.52, 1.02)	0.70 (0.50, 1.00)
	**Social support**										
	High	380 (62.7)	157 (41.3)	1	1	165 (43.4)	1	1	245 (64.5)	1	1
	Low	226 (37.3)	99 (43.8)	1.11 (0.79, 1.54)	1.20 (0.84, 1.70)	88 (38.9)	0.83 (0.59, 1.16)	0.85 (0.60, 1.19)	153 (67.7)	1.15 (0.81, 1.64)	1.20 (0.84, 1.72)
	**Job insecurity**										
	Low	322 (52.4)	118 (36.6)	1	1	141 (43.8)	1	1	209 (64.9)	1	1
	High	292 (47.6)	141 (48.3)	1.61 (1.17, 2.23)	1.51 (1.08, 2.12)	119 (40.8)	0.88 (0.64, 1.22)	0.86 (0.62, 1.19)	200 (68.5)	1.18 (0.84, 1.65)	1.13 (0.80, 1.59)
**CLERICAL WORKERS**	**Decision latitude**										
	High	245 (52.0)	94 (38.4)	1	1	101 (41.2)	1	1	160 (65.3)	1	1
	Low	226 (48.0)	116 (51.3)	1.69 (1.17, 2.44)	1.58 (1.07, 2.33)	101 (44.7)	1.15 (0.80, 1.66)	1.09 (0.75, 1.58)	157 (69.5)	1.21 (0.82, 1.78)	1.12 (0.75, 1.67)
	**Psychological demands**										
	Low	239 (49.3)	107 (44.8)	1	1	104 (43.5)	1	1	168 (70.3)	1	1
	High	246 (50.7)	114 (46.3)	1.07 (0.75, 1.52)	1.00 (0.68, 1.47)	105 (42.7)	0.97 (0.67, 1.38)	0.93 (0.64, 1.35)	159 (64.6)	0.77 (0.53, 1.13)	0.71 (0.48, 1.07)
	**Social support**										
	High	306 (64.4)	133 (43.5)	1	1	140 (45.8)	1	1	201 (65.7)	1	1
	Low	169 (35.6)	80 (47.3)	1.17 (0.80, 1.70)	1.24 (0.83, 1.85)	68 (40.2)	0.80 (0.55, 1.17)	0.80 (0.54, 1.18)	117 (69.2)	1.18 (0.79, 1.76)	1.19 (0.79, 1.80)
	**Job insecurity**										
	Low	232 (48.7)	88 (37.9)	1	1	107 (46.1)	1	1	150 (64.7)	1	1
	High	244 (51.3)	123 (50.4)	1.66 (1.15, 2.40)	1.60 (1.09, 2.34)	103 (42.2)	0.85 (0.59, 1.23)	0.83 (0.57, 1.19)	174 (71.3)	1.36 (0.92, 2.00)	1.31 (0.88, 1.94)

Abbreviations: OR: odds ratio; 95% CI: 95% confidence interval.

^a^ Sample size is not constant due to missing data in decision latitude (n = 46), psychological demands (n = 25), social support (n = 44) and job insecurity (n = 36).

^b^ The cut-off was the median of the symptom severity score (non-pain symptoms) obtained in the whole sample (median = 3.0; percentile 25th, percentile 75th = 2.0, 5.0).

^c^ The cut-off was the median of the widespread pain index (widespread pain) obtained in the whole sample (median = 0.0; percentile 25th, percentile 75th = 0.0, 1.0).

^d^ Adjusted for sex, education and occupational biomechanical demands.

Among all workers, jobs characterized by low decision latitude or high job insecurity were significantly associated to high levels of non-pain symptoms (adjusted OR (95% CI) = 1.50 (1.06, 2.11) and 1.51 (1.08, 2.12), respectively) but not related to high levels of widespread pain or multisite regional pain. Similar results were found when analysis was restricted to clerical workers, but with a slight increase in the magnitude of estimates (low decision latitude: adjusted OR = 1.58, 95% CI: 1.07, 2.33; high job insecurity: adjusted OR = 1.60, 95% CI: 1.09, 2.34).

**Fig 2:**
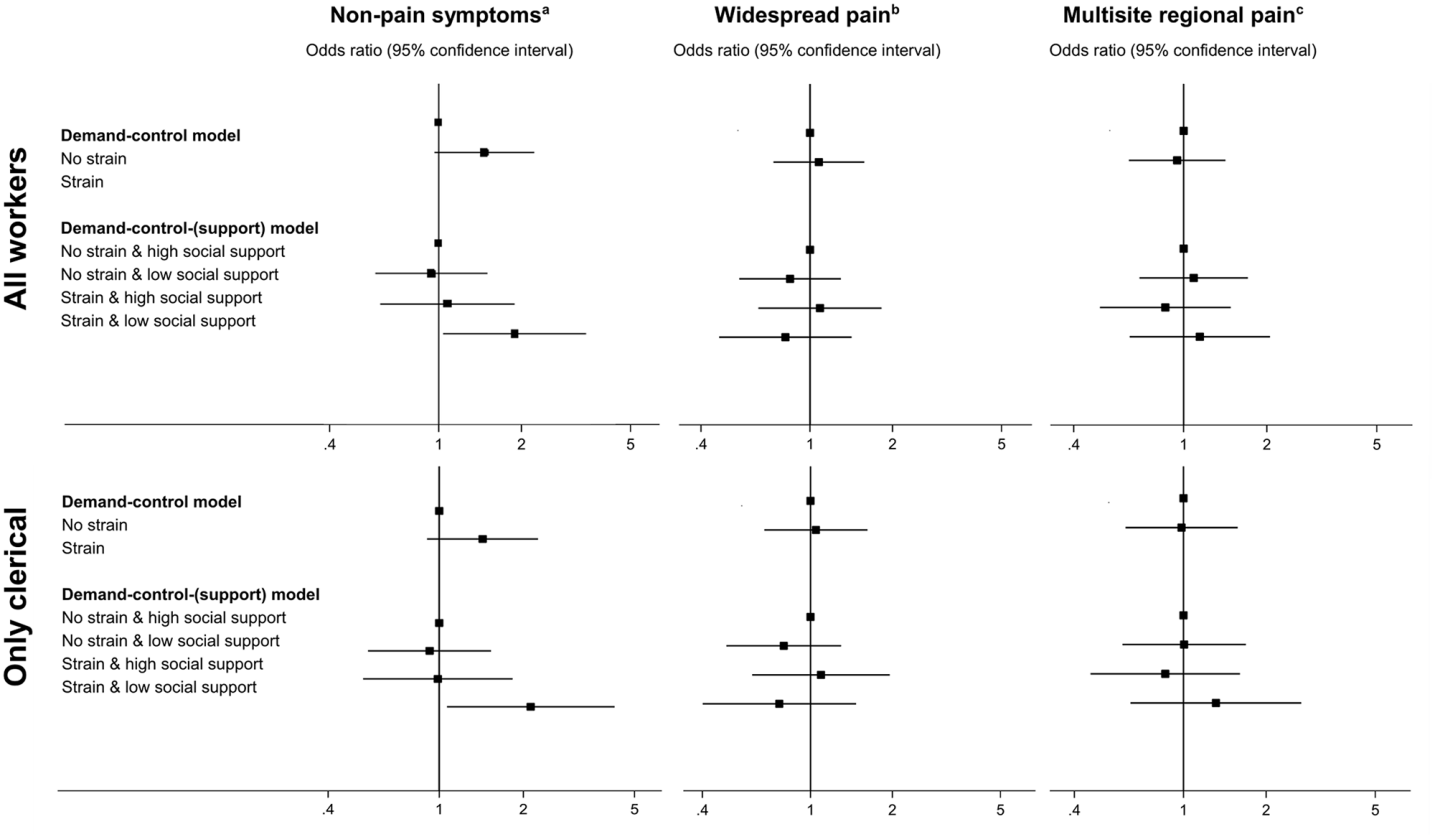
Associations between psychosocial work environment exposures (according to the demand-control and the demand-control-support models) and musculoskeletal outcomes (non-pain symptoms, widespread pain and multisite regional pain); odds ratios are adjusted for sex, education and occupational biomechanical demands and are presented for all workers and restricted to clerical workers (EPITeen cohort, 2011–2013). ^a^ The cut-off was the median of the symptom severity score (non-pain symptoms) obtained in the whole sample (median = 3.0; percentile 25th, percentile 75th = 2.0, 5.0). ^b^ The cut-off was the median of the widespread pain index (widespread pain) obtained in the whole sample (median = 0.0; percentile 25th, percentile 75th = 0.0, 1.0). ^c^ Multisite regional pain was considered present when 2 or more anatomical sites were reported as painful.

Fig 2 illustrates the effect of job strain (demand-control-support model) on musculoskeletal outcomes in all workers and restricted to those with clerical jobs.

Regarding non-pain symptoms, workers with strain jobs were significantly more likely to report high levels of this dimension of the widespread pain syndrome when compared with those with no-strain jobs. Although the statistical significance was borderline, this association was observed among all workers (adjusted OR = 1.45, 95% CI: 0.97, 2.16) as well as among clericals only (adjusted OR = 1.43, 95% CI: 0.91, 2.26). When social support was added to the main exposure, workers with strain jobs together with low social support were significantly more likely to score high in the non-pain dimension of the widespread pain syndrome when compared with those with no-strain jobs and high social support (all workers: adjusted OR = 1.86, 95% CI: 1.04, 3.31). This association was even stronger when only clerical workers were considered: adjusted OR = 2.12, 95% CI: 1.07, 4.24.

None of the factors included in the models of job strain (demand-control or demand-control-support) significantly predicted widespread pain or multisite regional pain.

## Discussion

In this population-based sample of young workers, jobs characterized by high strain were significantly associated with the non-pain dimension of the widespread pain syndrome and this association was strongly modified by social support. Nevertheless, these psychosocial work environment factors did not statistically predict widespread pain or multisite regional pain.

As a framework for the relation between work-related psychosocial factors and musculoskeletal health outcomes we used the demand-control-support model of job strain development [28]. This approach argues that psychological strain results not from a single aspect of the work environment, but from the joint effects of both the demands of a work situation and the range of decision-making freedom available to the worker facing those demands [29]. Adding to these occupational psychosocial vectors, this model emphasises that work-related social support may be crucial when the effect of job strain on health is being investigated [30], suggesting that a supportive relationship from/with supervisors and peers may contribute to decrease the negative impact of job strain on health outcomes [35, 36].

Presently, it is unequivocal that job strain is a major risk factor for the onset of work-related musculoskeletal complaints and that job social support may play an important role as an aggravator (low support) or attenuator (high support) of the impact of high-strain jobs on the musculoskeletal health of workers [37–40]. However, existing evidence has mainly originated from samples of long-term workers of specific occupational groups (e.g. nurses, mental health workers, factory workers) with clinically established disease [37], which dramatically reduces the opportunity for primary prevention or early intervention. Our study extends previous knowledge by examining the potential early effects of psychosocial context at the workplace in a sample of newly employed workers, who began their professional lives in the current context of employment flexibility, informality and insecurity.

In the EPITeen workers, we found increased non-pain complaints among those shortly exposed to high-strain and low social support jobs, which is in agreement with previous evidence on the negative impact of adverse psychosocial work environment on the health of workers [13]. Nevertheless, no significant associations were observed when musculoskeletal pain was the outcome.

To interpret the pattern of associations observed, we find three most likely explanations. First, the fact that we found statistical significant relations between psychosocial work environment and non-pain symptoms but not with pain complaints may be due to a true differential effect of the work exposure on different health outcomes. This is in line with the current understanding of the pathophysiology of the widespread pain syndrome, whereby it is expected that non-pain symptoms precede both multisite regional pain and widespread pain [14, 41, 42]. Our findings could be the result of the option to evaluate adults during what would be an early stage in the natural history of the condition, before pain symptoms are established, i.e., contrary to the non-pain symptoms, widespread and multisite regional pain are expected to represent more severe stages of musculoskeletal conditions that are more commonly observed among middle-aged and elderly subjects rather than in young adults [43]. In addition, the duration of exposure to work-related psychosocial stress among these young adult workers could still be too short to elicit more severe musculoskeletal symptoms such as those measured by the widespread pain score or through the presence of multisite regional pain.

A second explanation is that subjective constructs such as self-reported symptoms and work-related psychosocial factors are both influenced by individual psychological traits [44, 45]. This could originate spurious associations due to common determinants, which in fact have no causal interpretation with regard to the main effect being studied. Thirdly, we may hypothesize that reverse causation accounted for the observed associations, in that subjects with musculoskeletal complaints could tend to perceive the same work context as more strained or stressful than those without such symptoms. Notwithstanding, if confounding or reverse causation were the best explanations for our findings, we would also expect to observe the same pattern of significant associations with self-reported pain symptoms (regional and widespread), which did not happen.

Even though a plausible effect of psychosocial work environment on the onset of the widespread pain syndrome may be hypothesized since early stages of employment, the cross-sectional design of our study did not allow us to empirically confirm this model. The prospective follow-up of workers of the EPITeen cohort throughout their professional life will be fundamental for empirically confirming the etiological hypothesis regarding the effect of early exposures to work-related psychosocial stress on musculoskeletal outcomes.

Beyond what was already known on the impact of job strain on health outcomes, probably the most interesting finding of our work was that social support was an important modifier of the association between job strain and non-pain symptoms: young workers with strain jobs and low social support had twice the odds of reporting high levels of non-pain features than those with high-strain but high social support jobs. In such early stages of professional life, a supportive work environment may be especially important, since the need to share experiences, acquire new skills, and discuss difficulties is expected to be high [46]. Therefore, this research adds pioneer information on the potential role of social support in mitigating the negative impact of job strain on health outcomes.

Another important result was that job insecurity was related to high levels of the non-pain dimension of the widespread pain syndrome. The current flexibility and informality of occupations [5] have been selectively affecting young workers (unemployment, temporary and part-time jobs, low incomes, unstable works) [47, 48]. As the Portuguese rate of unemployment among young adults is high, workers of the EPITeen cohort (aged 21 years) are expected to be particularly exposed to adverse working conditions (e.g. insecure jobs, unstable and/or temporary jobs). Therefore, our finding may be a marker of the future potential population impact of job insecurity on early report of musculoskeletal complaints (non-pain symptoms).

To the best of our knowledge, this is the first study estimating the associations between high-strain and low social support jobs and the widespread pain syndrome dimensions in the beginning of professional life. These workers had the same chronological age, which eliminates confounding by birth cohort or calendar period. Moreover, the sample was population-based which adds a scenario of a wide range of real-world jobs performed in early career stages in a decade where the typology of employment has changed dramatically [49]. This is especially relevant since it adds updated knowledge on how young workers face the current labour market. Even though caution is needed regarding the generalizability of our findings to other young working populations from different countries and cultures, we do expect that some common features between European countries exist, namely those related to changing work typology, such as new jobs or decreased stability.

Some methodological issues need nevertheless to be addressed. Both psychosocial work environment and musculoskeletal symptoms may be influenced by several individual, social and cultural characteristics. Accordingly, all estimates were adjusted for a set of *a priori* defined confounders such as sex, education and occupational biomechanical demands. Data on contextual features such as ethnic background and cultural beliefs or interpersonal relationships and social capital were not collected in our study due to legal and logistic reasons, respectively. Although workers enrolled in our investigation were reasonably homogeneous regarding age and geographical origin (the proportion of immigrants in the metropolitan area of Porto was only 1.1% when the EPITeen cohort was assembled), residual confounding related to the surrounding context cannot be excluded.

In line with this, the presence of a chronic health condition among workers is also likely to influence both the perception of psychosocial work environment and the report of musculoskeletal symptoms. However, sensitivity analyses were performed by restricting estimates to workers without any chronic disease and similar conclusions to those found in the whole sample were supported by the results (results not shown).

Mental health status and related psychosocial problems may have changed the perception of the psychosocial work environment of workers. Nevertheless, in our sample, we did not observe significant associations between depressive symptoms (representing an important dimension of mental health status) and psychosocial work environment characteristics. Therefore, we believe that the mental dimension of health did not majorly impair the interpretation of our findings.

Since only 13.2% of workers (n = 77) had manual jobs, we did not have enough statistical power to conduct a stratified analysis in this group alone. Our option was to conduct a sensitivity analysis restricted to clerical workers (from whom we did have a larger sample size) in order to assess the potential impact of considering manual and clerical workers as a whole. As findings remained reasonably similar between the two approaches (all workers & only clerical workers), we believe that the inclusion of manual workers in our study did not largely affect the estimates. We cannot however exclude that our estimates may be indicating mainly what happens in clerical workers than in workers representing all types of jobs.

Health status may have conditioned the beginning of professional life of young adults, with healthier subjects entering in the labour market differentially. As workers were part of the EPITeen cohort, we were able to compare them with non-workers from the same source population regarding health conditions. We observed that the prevalence of chronic health conditions (proxy of health status) was reasonably similar between workers and non-workers, which suggests that selection bias for work due to health status did not occur. This was additionally supported by the fact that workers were more likely to report musculoskeletal complaints when compared with non-workers, which was not expected if the healthier young adults were overrepresented among workers.

In addition, the healthy worker effect, through which musculoskeletal symptoms may themselves determine the choice or persistence in a specific less strain job, cannot be excluded. Nevertheless, since most workers were recently employed (near 70% of them had been working for 12 months or less), it seems unlikely that musculoskeletal complaints had led to a substantial proportion of individuals to radically change their exposure status before the 21-year-old follow-up. Furthermore, the selection of a certain job depending on its level of strain is unlikely because psychosocial work environment features are particularly difficult to anticipate before starting on the job.

A reasonable fraction of workers enrolled in this study were unemployed. Although employed and unemployed workers were not significantly different regarding the main exposures and outcomes, a sensitivity analysis restricted to employed participants was conducted and the magnitude, direction and significance of the estimates remained similar to those found when all workers were considered (data not shown). Thus, we believe that work status did not represent a relevant source of bias to our findings.

As exposures and outcomes were collected cross-sectionally, it is not possible to discern the temporal sequence between the exposure to adverse work-related psychosocial factors and the manifestation of musculoskeletal complaints. Moreover, individuals with pain symptoms may have recalled psychosocial work environment exposure as more adverse and/or strained than those who did not present such complaints. However, data have been collected as part of an extensive protocol that assessed a wide spectrum of health-related information and subjects were not expected to be particularly aware of the main hypothesis addressed in this particular study.

Data on main exposures were collected using a single and self-reported source of information—the Job Content Questionnaire. Due to the fact that data were collected as part of the EPITeen population-based cohort (naturally working in a wide number of different companies), we were not able to objectively assess the psychosocial work environment *in loco*. Nevertheless, we believe that the impact of adverse psychosocial work environment characteristics on health outcomes might be more dependent on the individual perception and self-evaluation of the work-related psychosocial context than on the objectively-measured features of occupation [50]. Additionally, the Job Content Questionnaire is one of the most valid instruments to assess the overall psychosocial environment at the workplace [51].

The extent to which any cohort represents the source population throughout follow-up is likely to decrease over time because of differential losses to follow-up. Participants reassessed at 21 years old were reasonably similar to those lost to follow-up in a wide number of characteristics measured in the baseline evaluation, but attrition was significantly higher in individuals with low social position. Despite that, we do not believe that the underrepresentation of young adults with social disadvantage has substantially biased our estimates since we found no associations between parental education and musculoskeletal outcomes (non-pain symptoms, widespread pain and multisite regional pain) at 21 years of age.

Finally, as we were testing the associations between a wide spectrum of psychosocial work environment characteristics and musculoskeletal outcomes, multiple testing issues and consequent random effects may have occurred. However, the probability of random findings was expected to be low since all hypotheses were defined *a priori* according to the Karasek’s Job Strain Model [29]. Overall, most of the psychosocial work environment characteristics were significantly associated with non-pain symptoms but not with the remaining musculoskeletal outcomes in study, which was not expected if these effects were the result of multiple testing alone.

In conclusion, high-strain psychosocial work environments and job insecurity were significantly associated with the non-pain dimension of the widespread pain syndrome in the beginning of professional life. The potential effect of high strain was strongly modified by social support: workers who perceived their job as low socially supportive had twice the risk of reporting non-pain complaints.
